# Acrylamide toxic effects on mouse oocyte quality and fertility *in vivo*

**DOI:** 10.1038/srep11562

**Published:** 2015-06-25

**Authors:** Xing Duan, Qiao-Chu Wang, Kun-Lin Chen, Cheng-Cheng Zhu, Jun Liu, Shao-Chen Sun

**Affiliations:** 1 College of Animal Science and Technology, Nanjing Agricultural University, Nanjing 210095, China

## Abstract

Acrylamide is an industrial chemical that has attracted considerable attention due to its presumed carcinogenic, neurotoxic, and cytotoxic effects. In this study we investigated possible acrylamide reproductive toxic effects in female mice. Mice were fed an acrylamide-containing diet for 6 weeks. Our results showed the following effects of an acrylamide-containing diet. (1) Ovary weights were reduced in acrylamide-treated mice and oocyte developmental competence was also reduced, as shown by reduced GVBD and polar body extrusion rates. (2) Acrylamide feeding resulted in aberrant oocyte cytoskeletons, as shown by an increased abnormal spindle rate and confirmed by disrupted γ-tubulin and p-MAPK localization. (3) Acrylamide feeding resulted in oxidative stress and oocyte early stage apoptosis, as shown by increased ROS levels and p-MAPK expression. (4) Fluorescence intensity analysis showed that DNA methylation levels were reduced in acrylamide-treated oocytes and histone methylation levels were also altered, as H3K9me2, H3K9me3, H3K4me2, and H3K27me3 levels were reduced after acrylamide treatment. (5) After acrylamide feeding, the litter sizes of acrylamide-treated mice were significantly smaller compared to thus of control mice. Thus, our results indicated that acrylamide might affect oocyte quality through its effects on cytoskeletal integrity, ROS generation, apoptosis induction, and epigenetic modifications.

Acrylamide (ACR) is an important chemical that is used in various scientific and industrial processes, such as water treatment, in cosmetics, and for gel electrophoresis^1^. ACR is also found in carbohydrate rich foods that have been cooked at high temperatures, and in tobacco smoke^2^,^3^,^4^,^5^. In the US about 100,000 people may come in contact annually with ACR^6^. The lifelong exposure of these people to ACR at low intake levels through food and smoking has raised concerns regarding its potential health effects.

Previous studies have shown that ACR had negative effects on the thyroid^7^, spleen and erythrocytes, smooth muscle^8^, and skeletal muscle^9^. Additionally, as a neurotoxin, ACR may reduce nerve conduction velocity, causes rat hindlimb paralysis, increment of landing foot spread distance and dragging their feet^7^,^10^,^11^. Acrylamide adversely affected the cytoskeleton, particularly intermediate filaments^12^. Mice and rats which were administered ACR showed disrupted mating, reduced fertility rates, increased resumptions of fetuses, and sperm-head abnormalities in males^13^.

Oocyte quality can be monitored by the ability to mature, become fertilized, and give rise to normal offspring^14^. Oocyte maturation undergoes an asymmetric meiotic division, and extrudes a large haploid egg and a small polar body. During early meiosis I (MI), the meiotic spindle forms in the central oocyte and the chromosomes segregate to extrude the first polar body, which is essential for fertilization^15^. Previous work showed that ACR affected granulosa cell proliferation, the numbers of corpora lutea, and progesterone production in female mice^16^. However, the effects of ACR on oocyte quality are still poorly understood.

In present study we explored the effects of ACR on mouse oocyte maturation and its possible mechanism from oxidative stress, apoptosis and epigenetic modifications aspects. Oxidative stress was recently shown to be one of the key mechanisms involved in many chemically-induced cell injuries^17^. Oxidative stress can inhibit germinal vesicle break down and meiotic spindle assembly and function during oocyte maturation^18^,^19^. Additionally, increased reactive oxygen species (ROS) generation can alter several redox pathways and may ultimately result in apoptosis among oocytes and embryos^20^. While apoptosis is associated with normal ovarian development and function, such as prenatal germ cell death, granulosa cell death during postnatal follicular atresia, and ovarian surface epithelial cell death^21^,^22^. Epigenetic modifications of genomes are associated with two major mechanisms: DNA methylation and posttranslational modifications of histone proteins in chromatin. DNA methylation in oocytes is particularly significant, as it may play a key role in gene regulation and marks specific genes for activity in the embryo^23^. While disrupting histone modifications resulted in defective chromosome condensation and segregation, which delayed maturation progression^24^.

Our results showed the effects of ACR on oocyte developmental competence, cytoskeleton dynamics, ROS generation, early apoptosis, and epigenetic modifications, which provided evidence that ACR affected oocyte quality in an *in vivo* mouse model.

## Results

### Acrylamide effects on ovary weight and oocyte maturation

**Figure 1:**
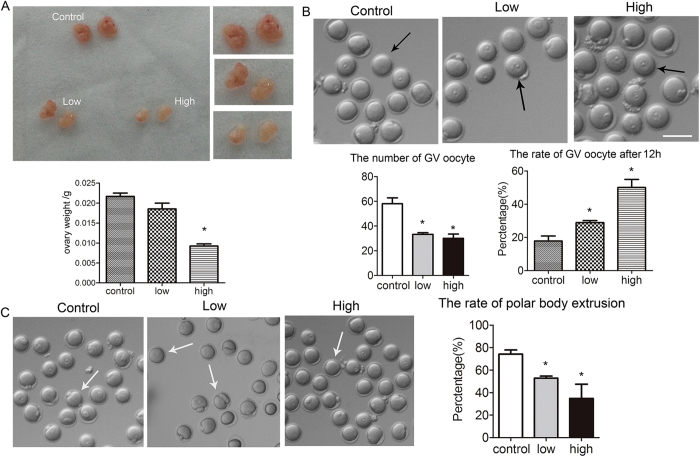
Acrylamide effects on ovary weight, numbers of GV oocytes, and developmental competence. **(A)** In acrylamide-treated mice, ovaries were significantly smaller and ovary weights were significantly reduced as compared to those of control mice. Mouse number = 5; error bar = SEM **(B)** After acrylamide treatment, the numbers of GV oocytes were reduced and GV rates were significantly increased after culture for 12 h. mouse number = 6, error bar = SEM, *p < 0.05. **(C)** After acrylamide treatment, the polar body extrusion rate was reduced after culture for 12 h. n = oocyte number; control: n = 334; low: n = 136; high: n = 88, error bar = SEM *p < 0.05. Bar = 150 μm.

To investigate acrylamide effects on mice, we examined for variations in their ovaries. In the acrylamide-treated groups, ovary sizes (Low: 0.0185 ± 0.00146 g; High: 0.00921 ± 0.00058 g) were clearly smaller than those in control mice (0.0217 ± 0.00087 g; Fig. 1A). Additionally, the numbers of oocytes obtained from ovaries were significant reduced after acrylamide treatment (Low: 33.3 ± 1.2; High: 30 ± 3.5) than those from control mice (58 ± 4.8; Fig. 1B).

Next, we examined the effect of acrylamide on oocyte developmental competence. After culturing for 12 hours, a large proportion of oocytes from treated mice remained in the GV stage and the percentages of GV oocytes were much higher (Low: 28.9 ± 1.2%, n = 197; High: 50.1 ± 4.9%, n = 209) than in control mice (17.8 ± 3.0% n = 308). Additionally, polar body extrusion by treated oocytes was reduced (Low: 52.9 ± 1.8%, n = 136; High: 34.9 ± 12.7%, n = 88) as compared to controls (74.3 ± 3.6%, n = 334, P < 0.05; Fig. 1C).

### Acrylamide effects on spindle organization in mouse oocytes

**Figure 2:**
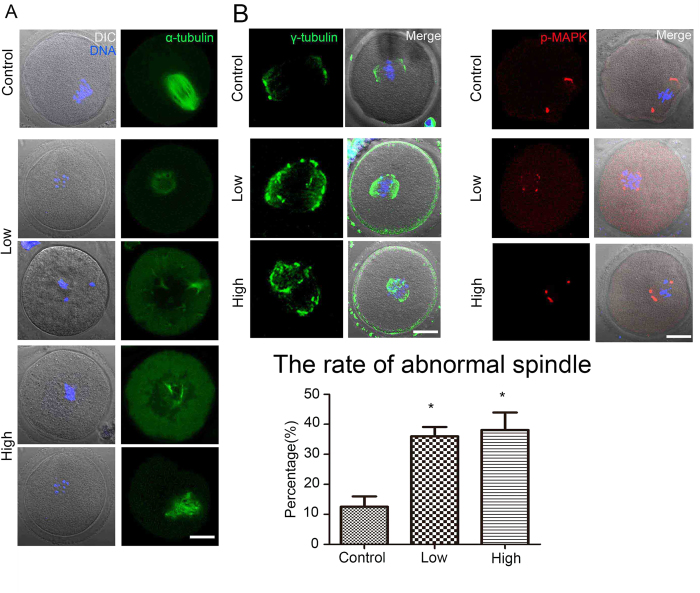
Acrylamide effects on oocyte spindle organization. **(A)** After acrylamide treatment, oocyte spindle organization was disrupted and the rate of abnormal spindle formation was significantly increased. Mouse number = 6; n = oocyte number; control: n = 255; low: n = 140; high: n = 87, error bar = SEM **(B)** Localization of γ-tubulin and p-MAPK. After acrylamide treatment, the localization of γ-tubulin and p-MAPK in oocytes were disrupted. Green: spindle; Blue: chromatin; Red: p-MAPK. *p < 0.05. Bar = 20 μm.

To investigate why the oocytes in acrylamide-treated mice exhibited reduced developmental competence, we examined the effects of acrylamide on cytoskeletal integrity in mouse oocytes by assessing meiotic spindle organization. In the control group, most oocytes showed normal spindle morphologies in the MI stage, whereas after acrylamide treatment, a large proportion of oocytes had disrupted spindles with significantly higher abnormal spindle rates (Low: 36 ± 3.4%, n = 140; High: 38.1 ± 5.8%, n = 87) than in the controls (12.5 ± 3.4%, n = 255, P < 0.05; Fig. 2A). Additionally, γ-tubulin and p-MAPK localizations were disrupted in treated oocytes. These results indicated that acrylamide had disrupted oocyte cytoskeletal integrity (Fig. 2B).

### Acrylamide-induced reactive oxygen species (ROS) generation in mouse oocytes

**Figure 3:**
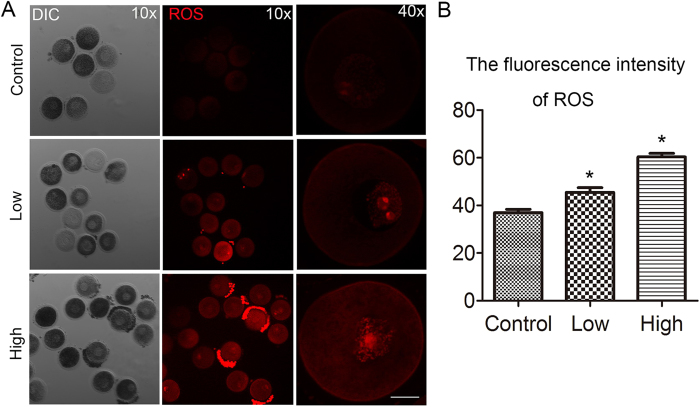
Acrylamide effects on ROS generation in GV oocytes. **(A)** Acrylamide effect on ROS generation determined by DHE fluorescence in GV oocytes. **(B)** After acrylamide treatment, fluorescence intensity was significantly increased. Mouse number = 6; n = oocyte number, control: n = 41, low: n = 41, high: n = 41, error bar = SEM, Red, ROS; *p < 0.05. Bar = 20 μm.

To further examine the effects of acrylamide on mouse oocytes, we investigated intracellular ROS generation. As shown in Fig. 3A, compared to the control group, acrylamide treatment resulted in a dramatic increase in ROS generation in mouse oocytes. ROS fluorescence intensity was significantly higher with acrylamide treatment (high: 60.3 ± 1.5, n = 41; low: 45.5 ± 1.9, n = 41) than in control oocytes (36.9 ± 1.4, n = 41, P < 0.05; Fig. 3B), which indicated that increased oxidative stress had occurred after acrylamide treatment.

### Acrylamide-induced apoptosis among mouse oocytes

**Figure 4:**
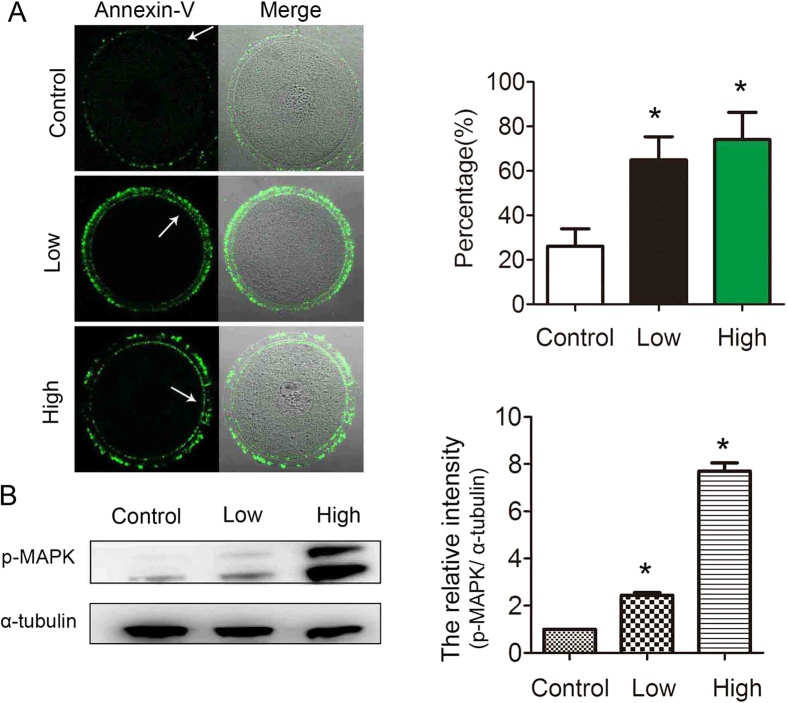
Acrylamide effects on GV oocyte apoptosis. **(A)** Acrylamide induced early-stage apoptosis among GV oocytes. In control oocytes, normal oocytes exhibited fluorescence on the zona, whereas oocytes in early apoptosis exhibited fluorescence on the zona and oocyte membrane. The early apoptosis rate in the acrylamide-treated group was significantly increased. Mouse number = 6; n = oocyte number, control: n = 89, low: n = 54, high: n = 61, error bar = SEM **(B)** Cropped gels were used for our western blot results. Western blot showed that the apoptosis-related protein p-MAPK expression was increased in the acrylamide group. p-MAPK molecular mass is 42/44 kDa and that of α-tubulin is 52 kDa. Relative p-MAPK and α-tubulin staining intensity were assessed by densitometry. *p < 0.05. Bar = 20 μm.

Apoptosis plays a major role in determining cell fate. Thus, we assessed apoptosis occurrence among mouse oocytes. As shown in Fig. 4A, Annexin-V staining showed that control oocytes were viable and non-apoptotic with green fluorescent signals only at the zona pellucida. In contrast, after acrylamide treatment, GV oocytes showed clear green signals in the oocyte membrane and zona pellucida, which indicated that acrylamide-treated had resulted in early apoptosis among mouse oocytes. The early apoptosis rate of GV oocytes with acrylamide treatment was significantly higher (Low: 64.9 ± 10.4%, n = 54; High: 74.1 ± 12.3%, n = 61) than that of controls (26.1 ± 7.8%, n = 89). To further confirm this, we assessed the expression of apoptosis related proteins.

Western blot results showed that the apoptosis-related proteins p-MAPK (1 vs. 2.44 vs. 7.71 for current band, P < 0.05) expression was significantly increased after acrylamide treatment as compared with those of controls (Fig. 4B).

### Acrylamide effects on DNA methylation and histone methylation levels in mouse oocytes

**Figure 5:**
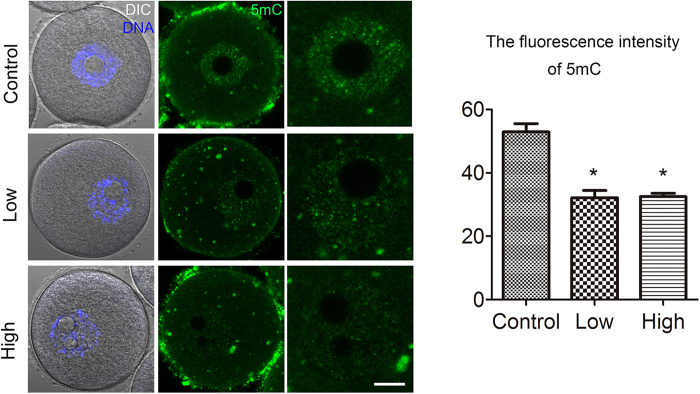
Acrylamide effects on DNA methylation in mouse oocytes. **(A)** Immunofluorescent staining for 5 mC in mouse oocytes. Fluorescence intensity was significantly reduced in the acrylamide-treated group. Mouse number = 6; n = oocyte number, control: n = 15, low: n = 15, high: n = 15, error bar = SEM *p < 0.05. Bar = 20 μm.

We next investigated possible acrylamide effects on epigenetic modifications in mouse oocytes. We first assessed DNA methylation levels by immunofluorescent staining. As shown in Fig. 5, in the acrylamide treated groups, 5mC fluorescence intensity was significantly reduced (Low: 32.2 ± 2.3, n = 15; high: 32.5 ± 1.0, n = 15) as compared with that in controls (52.9 ± 2.6, n = 15, P < 0.05).

**Figure 6:**
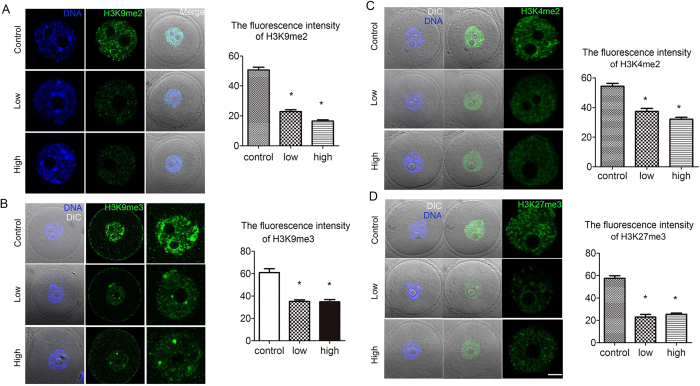
Acrylamide effects on histone methylation in mouse oocytes. **(A,B)** Immunofluorescent staining for H3K9me2 and H3K9me3 in mouse oocytes. H3K9me2 and H3K9me3 levels were significantly lower in the acrylamide-treated group. Mouse number = 6; n = oocyte number, n(H3K9me2) = 25; n(H3K9me3) = 23, error bar = SEM **(C,D)** Immunofluorescent staining for H3K4me2 and H3K27me3 in mouse oocytes. Fluorescence intensity levels of H3K4me2 and H3K27me3 were significantly reduced in the acrylamide-treated group. Mouse number = 6; n = oocyte number, n(H3K4me2) = 25; n(H3K27me3) = 21, error bar = SEM *p < 0.05. Bar = 20 μm.

With regard to histone methylation modifications, in treated oocytes, the levels of H3K9me2 (22.9 ± 1.2 and 16.5 ± 0.8 versus 50.7 ± 1.9, n = 25) and H3K9me3 (35.2 ± 1.3 and 34.7 ± 2.0 versus 60.9 ± 3.5, n = 23 P < 0.05) were significantly reduced as compared with those in controls. A quantitative analysis showed that the fluorescence intensities of H3K9me2 and K3K9me3 were significantly lower than those in controls (Fig. 6A,B).

We also examined H3K4 and H3K27 methylation levels. As shown in Fig. 6C, in treated oocytes, H3K4me2 expression was significantly reduced (Low: 37.4 ± 2.0, n = 25; High: 32.1 ± 1.3, n = 25) as compared with that in controls (54.3 ± 1.9, n = 25, P < 0.05). The H3K27me3 level was also lower in treated oocytes (Low: 22.9 ± 2.4, n = 21; High: 25.4 ± 1.1, n = 21) than that in controls (57.5 ± 2.3, n = 21, P < 0.05; Fig. 6D).

### Acrylamide effects on mouse litter size

**Figure 7:**
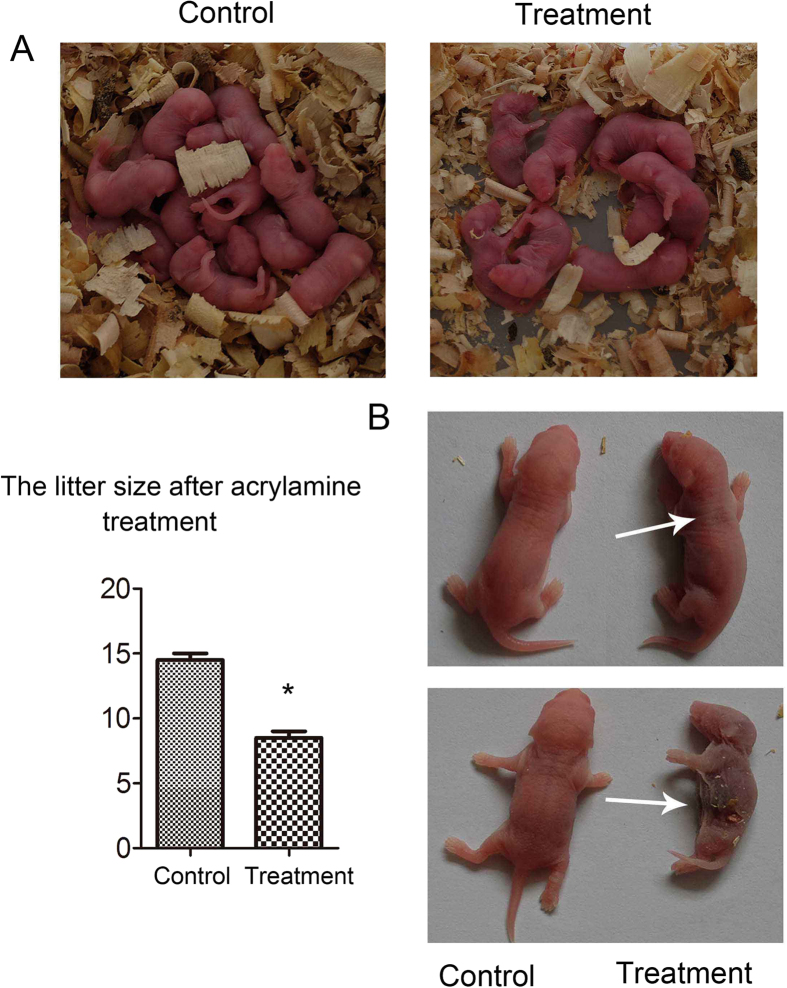
Acrylamide effects on mouse litter size. **(A)** Offspring numbers were significantly lower after acrylamide treatment as compared to control mice. **(B)** Acrylamide effects on offspring gross appearance. *p < 0.05 error bar = SEM.

We also examined mouse offspring after acrylamide treatment. For acrylamide-treated mice, the average litter size was significantly smaller (8.5 ± 0.5) as compared to control mice (14.5 ± 0.5, P < 0.05; Fig. 7A). In addition, for treated mice, some of their offspring had a black body and some died after 2 days (Fig. 7B).

## Discussion

In this study, we investigated acrylamide effects on mouse oocytes by assessing oocyte quality, oxidative stress, oocyte apoptosis, epigenetic modifications, and mouse litter sizes. Our results suggested that feeding mice an acrylamide contaminated diet had toxic effects on the oocytes.

A previous study clearly demonstrated that oral administration of acrylamide to male mice resulted in a significant reduction in their body and organ weights^13^. This was consistent with our results with female mice which indicated that acrylamide might have toxic effects on the female reproductive system due to reduced ovary weights and numbers of GV oocytes. Acrylamide was shown to affect follicular development and corpus luteum formation^16^, and we focused on the effects of acrylamide on the quality of mouse oocytes, since oocyte quality is critical for oocyte maturation, fertilization, and embryo quality^25^. Our results showed that big proportion of oocytes could not enter meiosis, and the rate of polar body extrusion was significantly reduced in acrylamide-treated oocytes. These results indicated that acrylamide exposure had reduced the developmental competence of oocytes.

To further determine the effects of acrylamide on oocyte quality, we examined spindle organization during mouse oocyte maturation. It was reported that acrylamide disrupted the function of kinesin-related proteins and also disrupted microtubule assembly in mitosis^26^. Our results showed that a large proportion of oocytes had aberrant meiotic spindle morphologies, which was consistent with the results in the previous study. Additionally, the disrupted localization of γ–tubulin and p-MAPK, a centrosome protein that regulates spindle formation, further confirmed the damage to spindles^27^. This spindle morphology disruption further demonstrated that acrylamide had a toxic effect on mouse oocyte quality.

In biological systems, ROS are key signaling molecules in various physiological processes, such as meiotic resumption, cellular apoptosis, and senescence^28^,^29^. Moreover, mammalian oocytes and embryos are extremely sensitive to ROS and a slightly increased ROS level can disrupt oocyte maturation and embryo development, which promotes embryo fragmentation^30^. Our results showed that acrylamide contributed to excessive ROS generation in GV oocytes, which was one reason for oocyte maturation failure. It was reported that higher ROS levels could alter several redox pathways and may ultimately result in apoptosis among oocytes and embryos^20^. Other recent studies also showed that acrylamide could induce apoptosis in testes, bovine lens epithelial cells, neuroblastoma cells, human promyelocytic leukemia cells, and astrocytoma cells^31^,^32^,^33^,^34^,^35^. Annexin-V, a phospholipid-binding protein, is used to detect the translocation of the phospholipid phosphatidylserine (PS) from the inner to the outer cytoplasmic membrane, which is known to occur during the early stage of apoptosis^36^. Our results suggested that acrylamide caused early apoptosis among mouse oocytes, showing with the high level of Annexin-V. MAPK signaling pathway has also been found to be induced in response to apoptosis^37^, and increased p-MAPK expression further confirmed that acrylamide caused oocyte apoptosis. These results indicated that acrylamide may induce the oxidant stress related apoptotic death of mouse oocytes.

In order to examine the general and overall methylation level, we used immunofluorescent staining to determine epigenetic modification rather than combined bisulfite restriction analysis (COBRA) and bisulfite sequencing. DNA methylation is a well-characterized epigenetic modification that is crucial for normal mammalian development, retrotransposon silencing, and cellular reprogramming. DNA methylation status is established during gametogenesis and early embryo development^38^. Previous studies showed that some DNA methylation patterns established in oocytes left a vitally important imprint in the embryo. Additionally, increases in epigenetic modifications are related to oocyte meiotic and developmental competence during oocyte maturation^39^,^40^. The reduced 5mC level we found showed that acrylamide affected oocyte DNA methylation status by modifying 5mC level.

The transition from a non-surrounded nucleolus to a surrounded nucleolus configuration is critical for acquiring full developmental competence by oocytes^41^. During this process, H3K9 methylation levels were higher in SN-type mouse oocytes than in NSN-type oocytes^42^. The reduced H3K9me2/3 level we found indicated that acrylamide might affect the chromatin configuration in oocytes by altering the H2K9me2/3 levels. Methylation of lysine residue 27 of histone H3 (H3K27), an epigenetic mark, is closely linked to transcriptional repression^43^. During embryonic stem cell differentiation, H3K27me3 plays an essential role in silencing the expression of key developmental genes^44^. Our results showed that acrylamide significantly reduced H3K27me3 expression, which suggested that acrylamide might alter the transcriptional activity of the oocyte genome. While trimethylation of lysine 4 at histone 2 (H3K4me2) is associated with active transcription, as it plays an important role in gene expression^45^. Thus, an altered H3K4me2 level might affect the maturation of oocytes, and our results showed that acrylamide apparently reduced H3K4me2 levels, which indicated that acrylamide also altered the transcriptional activity of the oocyte genome by mediating H3K4me2 expression.

A recently study showed that acrylamide might induce oocyte apoptosis, particularly in primordial follicles and primary follicles, and reduced the numbers of ovarian follicles in newborn guinea pig offspring^46^. Our results showed that after acrylamide treatment of female mice, the number of offspring was significantly reduced, which further confirmed the effect of acrylamide on oocyte quality and offspring production.

In conclusion, our results indicated that acrylamide might have effects on the female reproductive system by affecting oocyte quality, cytoskeletal integrity, ROS levels, apoptosis induction, epigenetic modifications, and mouse litter sizes.

## Materials and Methods

### Animals and feeding regimens

All procedures with mice were conducted according to the Animal Research Institute Committee guidelines of Nanjing Agriculture University, China. The experimental protocols were approved by Nanjing Agriculture University Animal Research Institute Committee. Mice were housed in a temperature controlled room with appropriate dark-light cycles, fed a regular diet, and maintained under the care of the Laboratory Animal Unit, Nanjing Agricultural University, China. Mice were randomly assigned to 3 groups (mice number = 40/group), and acrylamide was dissolved in water. The acceptable daily intake (ADI) of acrylamide for these groups was 0 mg/kg/d (control mice), 10 mg/kg/d, and 50 mg/kg/d according to the previous work^47^,^48^,^49^. Female 3-week-old ICR mice were kept at a constant temperature of 24 ± 2 °C on a 12 h light/dark cycle and had unrestricted access to food and water throughout the study period that lasted 6 weeks.

Germinal vesicle-intact oocytes were harvested from ovaries and cultured in M16 medium (Sigma, MO) under paraffin oil at 37 °C in a 5% CO_2_ atmosphere. Oocytes were used at different culture times for immunofluorescent staining and Western blot analysis.

### Antibodies and chemicals

A mouse monoclonal anti-α-tubulin-FITC antibody was from Sigma (St Louis, MO). Alexa Fluor 488, 594 antibodies were from Invitrogen (Carlsbad, CA). Rabbit polyclonal anti-γ-tubulin. Rabbit polyclonal anti-p-MAPK was from Cell Signaling Technology (Danvers, MA). Mouse monoclonal anti-5mC antibody and rabbit polyclonal anti-H3K9me2/3 antibody were from Abcam (Cambridge, UK). Rabbit monoclonal anti-H3K27me3 antibody and rabbit polyclonal anti-H3K4me2 antibody were from Cell Signaling Technology (Danvers, MA, USA).

### Confocal microscopy

For single staining for γ-tubulin, spindles, oocytes were fixed in 4% paraformaldehyde in PBS at room temperature for 30 min and then transferred to membrane permeabilization solution (0.5% Triton X-100) for 20 min. After 1 h in blocking buffer (1% BSA-supplemented PBS), oocytes were incubated at 4 °C overnight or at room temperature for 4 h with rabbit anti-γ-tubulin (1:100). After three washes in a wash buffer (0.1% Tween 20 and 0.01% Triton X-100 in PBS), oocytes were labeled with Alexa Fluor 594 goat-anti-rabbit IgG (1:100; for γ-tubulin staining) at room temperature for 1 h. These specimens were co-stained with Hoechst 33342 for 10 min and then washed three times in wash buffer. Specimens were mounted on glass slides and examined with a confocal laser-scanning microscope (Zeiss LSM 700 META). At least 30 oocytes were examined for each group.

### Fluorescence intensity analysis

Fluorescence intensity was assessed using Image J software (NIH). For fluorescence intensity analysis, samples of control and treated oocytes were mounted on the same glass slide. And we used the same parameters to normalize across replicates. After immunofluorescent staining, the average fluorescence intensity per unit area within the region of interest (ROI) of immunofluorescence images was examined. Independent measurements using identically sized ROIs were taken for the cell cytoplasm. When calculating the fluorescence intensity, we ignored the abnormal ones (little oocytes with extreme strong or weak). Average values of all measurements were used to determine the final average intensities for control and treated oocytes.

### ROS products

To determine the amount of ROS generation, GV oocytes were loaded with the oxidation-sensitive fluorescent probe dihydroethidium (DHE; Beyotime Institute of Biotechnology, Hangzhou, China) by incubation at 37 °C for 30 min in DPBS and then mounted on glass slides. Fluorescent signals were measured using a confocal microscope (Zeiss LSM 700). Photographs were analyzed using Image J software (Research Services Branch, National Institute of Mental Health, Bethesda, MD, USA) to measure brightness for each oocyte.

### Oocyte Annexin-V staining

Oocytes were stained using an Annexin-V staining kit (Beyotime Institute of Biotechnology Hangzhou, China), according to the manufacturer’s instructions. Briefly, live oocytes were washed twice in PBS and stained for 10 min in the dark with 195 μL of binding buffer, which contained 5 μL of Annexin-V-FITC. Fluorescent signals were measured using a confocal microscope (Zeiss LSM 700).

### Western blot analysis

A total of 150 mouse oocytes were placed in Laemmli sample buffer (SDS sample buffer with 2-mercaptoethanol) and heated at 100 °C for 5 min. Proteins were separated by SDS-PAGE and then electrophoretically transferred to PVDF membranes. Membranes were blocked with PBST (PBS containing 0.1% Tween 20) that contained 5% non-fat milk for 1 h, followed by incubation at 4 °C overnight with a rabbit monoclonal anti-p-MAPK (1:1000) and a rabbit monoclonal anti-β-actin or tubulin antibody (1:2000; for p-MAPK, incubation buffer was 5% BSA in PBST). After washing 3 times in PBST (10 min each), membranes were incubated at 37 °C for 1 h with HRP-conjugated Pierce Goat anti-Rabbit IgG (1:10,00). Finally, membranes were processed using an enhanced chemiluminesence detection system (Amersham, Piscataway, NJ).

### Statistical analysis

At least three biological replicates were used for each analysis. Each replicate was done by an independent experiment at the different time. Results are given as means ± SEM. Statistical comparisons were made using analysis of variance (ANOVA) and differences between treatments groups were assessed with Duncan’s multiple comparisons test. A p-value of <0.05 was considered significant.

## Additional Information

**How to cite this article**: Duan, X. *et al*. Acrylamide toxic effects on mouse oocyte quality and fertility *in vivo*. *Sci. Rep*. **5**, 11562; doi: 10.1038/srep11562 (2015).

## Supplementary Material

Supplementary Information
