# Supplementary material for: Acrylamide toxic effects on mouse oocyte quality and fertility in vivo
Source: Sci Rep. 2015 Jun 25;5:11562. doi: 10.1038/srep11562 (PMC4479821; doi:10.1038/srep11562)

# Acrylamide toxic effects on mouse oocyte quality and fertility *in vivo*

Xing Duan, Qiao-Chu Wang, Kun-Lin Chen, Cheng-Cheng Zhu, Jun Liu, Shao-Chen Sun^*^

College of Animal Science and Technology, Nanjing Agricultural University, Nanjing 210095, China.

**^*^Correspondence to:** Shao-Chen Sun, College of Animal Science and Technology, Nanjing Agricultural University, Nanjing, China. Tel/Fax: +86-25-84399092. E-mail: [sunsc@njau.edu.cn](mailto:sunsc@njau.edu.cn)

Supplementary figure: The original figure for the bands of p-MAPK (42/44 kDa), α-tubulin (52kDa) and the band marker for the western blot experiments.

###
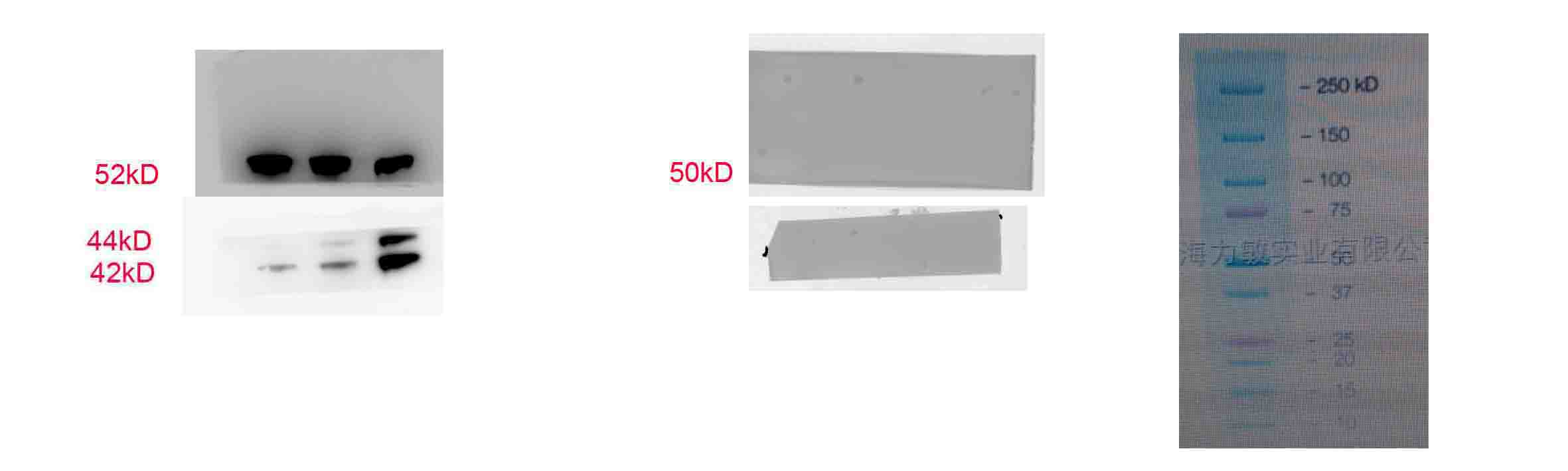

Supplement: Supplementary Information [file srep11562-s1.doc]
